# Silencing of the Mycorrhiza-Inducible Phosphate Transporter *TaPT3-2D* in Wheat Enhances Pathogen Susceptibility and Impairs Arbuscular Mycorrhizal Symbiosis

**DOI:** 10.3390/plants15010118

**Published:** 2026-01-01

**Authors:** Yi Zhang, Danfeng Wang, Yuchen Ma, Xueqing Wang, Kedong Xu, Xiaoli Li, Xinxin Shangguan, Haohao Cao, Guozhang Kang, Chengwei Li

**Affiliations:** 1 Key Laboratory of Plant Genetics and Molecular Breeding, Zhoukou Normal University, Zhoukou 466001, China; zyzknu2009@163.com; 2 Henan Key Laboratory of Crop Molecular Breeding and Bioreactor, Zhoukou Normal University, Zhoukou 466001, China; 3 Henan Plant Gene and Molecular Breeding Engineering Research Center, Zhoukou 466001, China; wdf19911025@live.com (D.W.); 17637321715@163.com (Y.M.); xueqingwang4011@163.com (X.W.); 4 Henan Crop Molecular Design Breeding and Cultivation Engineering Technology Research Center, Zhoukou 466001, China; xukd1107@126.com (K.X.); xiaoli890107@163.com (X.L.); shangguanxin0409@163.com (X.S.); haohaocao@126.com (H.C.); 5 The State Key Laboratory of High-Efficiency Production of Wheat-Maize Double Cropping, Henan Agricultural University, Zhengzhou 450046, China; 6 School of Agricultural Sciences, Zhengzhou University, Zhengzhou 450001, China

**Keywords:** phosphate transporter, *Triticum aestivum* L., disease susceptibility, virus-induced gene silencing, Pi uptake, mycorrhizal symbiosis

## Abstract

The interplay between phosphate (Pi) signaling and defense pathways is crucial for plant fitness, yet its molecular basis, particularly in wheat, remains poorly understood. Here, we functionally characterized the plasma membrane-localized high-affinity phosphate transporter TaPT3-2D and demonstrated its essential roles in Pi uptake, arbuscular mycorrhizal (AM) symbiosis, and fungal disease resistance. Quantitative analyses showed that *TaPT3-2D* expression was strongly induced by AM colonization (165-fold increase) and by infection with *Bipolaris sorokiniana* (54-fold increase) and *Gaeumannomyces tritici* (15-fold increase). In contrast, virus-induced gene silencing (VIGS) of *TaPT3-2D* reduced Pi uptake and mycorrhizal colonization. Moreover, *TaPT3-2D*-silenced plants exhibited increased susceptibility to biotrophic, hemibiotrophic, and necrotrophic fungi, accompanied by reduced expression of pathogen-related genes. The simultaneous impairment of Pi uptake, AM symbiosis, and defense responses in silenced plants indicates that TaPT3-2D functionally couples these processes. Functional complementation assays in low-Pi medium further revealed that *TaPT3-2D* partially rescued defective Pi uptake in mutant MB192 yeast, supporting its role as a high-affinity phosphate transporter. Collectively, these results identify *TaPT3-2D* as both a key regulator of individual pathways and as a molecular link connecting Pi homeostasis, symbiotic signaling, and disease resistance in wheat.

## 1. Introduction

As an essential macronutrient, inorganic phosphate (Pi) availability frequently limits the productivity of major cereal crops such as wheat, posing a significant challenge to global food security [1]. This limitation arises from its rapid fixation into insoluble complexes within the soil matrix, inherently low bioavailability, and spatially heterogeneous distribution in natural soil environments [2]. In response, plants have evolved a range of adaptive strategies to optimize Pi acquisition. These include (i) remodeling root system architecture to exploit heterogeneous soil Pi distributions; (ii) secreting acid phosphatases and phytases to hydrolyze organic phosphate esters and mobilize fixed Pi; (iii) establishing arbuscular mycorrhizal (AM) symbioses to enhance Pi absorption via fungal hyphae; and (iv) inducing high-affinity phosphate transporters to facilitate Pi uptake under low-Pi conditions [2].

AM symbioses are evolutionarily conserved mutualistic relationships between Glomeromycotina fungi and more than 70% of extant vascular plant species [3,4]. Functional AM symbioses require host plants to allocate substantial amounts of photosynthetically derived carbon in the form of hexoses and C16:0 β-monoacylglycerol to intraradical fungal structures, sustaining fungal growth and development [5]. Reciprocally, AM fungi efficiently forage for and redistribute water and inorganic nutrients (e.g., Pi and nitrate) through extensive extraradical hyphal networks. These resources are delivered to host roots across symbiotic interfaces via specialized membrane-embedded transporter complexes [5].

Following host root penetration, intraradical hyphae traverse outer cortical cell layers and undergo extensive dichotomous branching to form specialized arbuscules within inner cortical cells [3]. These ephemeral branched structures establish dedicated symbiotic interfaces that facilitate bidirectional molecular signaling during symbiosis establishment and high-efficiency nutrient exchange during the mature arbuscular stage [4]. During arbuscule development, a plant-derived peri-arbuscular membrane (PAM) forms concentrically around the branching fungal hyphae, establishing a physical barrier that segregates the fungal symbiont from the host cytosol and concurrently functions as a dedicated symbiotic resource exchange interface [6]. The PAM is enriched in high-affinity proton-coupled phosphate transporters (predominantly PHT1-family proteins), which are strictly required for unidirectional Pi transfer from the fungus to the plant host [6,7].

The concerted transcriptional induction of multiple phosphate transporters constitutes a core adaptive response to Pi starvation in higher plants [8]. These plasma membrane-localized high-affinity transporters are rapidly upregulated under low-Pi conditions and serve as the primary mediators of Pi acquisition and redistribution, thereby maintaining systemic Pi homeostasis [8,9]. PHT1 orthologs have been identified in numerous plant species, including nine in *Arabidopsis thaliana* and 13 in *Oryza sativa* [10,11]. In *A. thaliana*, AtPT1, AtPT4, AtPT5, AtPT8, and AtPT9 perform distinct roles in Pi absorption and long-distance transport [12,13,14,15]. In rice, OsPT2, OsPT6, OsPT9, and OsPT10 predominantly mediate high-affinity Pi uptake from the rhizosphere during Pi deprivation [16,17]. Notably, the expression of *OsPT9* and *OsPT10* appears to be largely independent of Pi availability, enabling sustained Pi uptake under Pi-replete conditions and contributing to Pi homeostasis [16]. The allohexaploid bread wheat (*Triticum aestivum* L.) genome consists of three homoeologous subgenomes (A, B, and D) and therefore contains many homoeologous gene copies [18]. Consistent with this genomic complexity, wheat harbors an expanded phosphate transporter gene family comprising 35 identified members [19]. Among these, overexpression of *TaPHT1;4* and *TaPT2* significantly enhances rhizosphere Pi acquisition efficiency and promotes biomass accumulation [20,21]. In addition, *TaPHT1.9-4B* coordinates Pi uptake, long-distance Pi translocation, and growth under Pi starvation [22].

The essentiality of phosphorus for plant growth is undisputed. Accordingly, Pi uptake is tightly regulated to maintain optimal cellular Pi status while balancing colonization by beneficial microbes with constitutive defense readiness and inducible immune responses against fungal pathogens [23,24,25,26]. The acquisition of adequate Pi not only supports normal growth and development but also enhances pathogen defense capacity [23]. For example, foliar application of 50 mM K_2_HPO_4_ to rice increased grain yield by up to 32% while reducing blast disease (*Magnaporthe oryzae*) incidence by up to 42% [24]. However, plant defense responses under different Pi regimes are highly variable and depend on genetic background, as well as crosstalk between Pi availability and key defense hormone pathways, including salicylic acid (SA), jasmonic acid (JA), and ethylene signaling [25,26,27,28]. In rice, both excessive Pi fertilization and Pi overaccumulation repress the expression of defense-related genes, thereby compromising resistance to *M. oryzae* compared with sufficient- or low-Pi conditions [25]. In contrast, in *A. thaliana*, elevated Pi levels are associated with increased resistance to necrotrophic (*Plectosphaerella cucumerina*) and hemibiotrophic (*Colletotrichum higginsianum*) fungal pathogens [26,27]. Finally, phosphorus deficiency can suppress plant immunity, potentially favoring associations with plant growth-promoting rhizobacteria (PGPR) that enhance Pi acquisition efficiency and/or alleviate Pi stress [28]. Together, these findings highlight the importance of tightly regulated Pi uptake in maintaining cellular Pi homeostasis while balancing microbial interactions with effective defense against fungal pathogens.

Although the phosphate transport functions of PHT1 family members are well characterized, their direct involvement in immune signaling, especially within symbioses, remains poorly understood. We previously found that six *TaPHT1* genes, including the newly identified *TaPT3-2D*, are constitutively upregulated in AM-colonized wheat roots regardless of external Pi status [19]. This Pi-independent expression suggests that *TaPT3-2D* regulation may be linked to symbiotic or defense signaling rather than nutrient sensing alone, raising the possibility that it integrates multiple biotic signals. To test this hypothesis, we conducted a functional analysis of *TaPT3-2D*, focusing on its subcellular localization, biochemical activity as a phosphate transporter, and physiological roles in plant-microbe interactions and Pi homeostasis. Compared with wild-type (WT) plants, *TaPT3-2D*-silenced wheat exhibited increased susceptibility to biotrophic, hemibiotrophic, and necrotrophic fungal pathogens; impaired AM symbiosis; and reduced Pi accumulation. Together, these results indicate that the mycorrhiza-inducible phosphate transporter TaPT3-2D regulates wheat immunity, AM symbiosis, and Pi uptake.

## 2. Results

### 2.1. TaPT3-2D Is an AM-Inducible Phosphate Transporter Gene

We previously performed a genome-wide characterization of the wheat *PHT1* gene family and identified *TaPT3-2D* as a novel AM-inducible gene. Sequence analysis in Chinese Spring wheat revealed that *TaPT3-2D* maps to the chromosomal locus *TraesCS2D02G045700*. In addition, the two homologous genes *TraesCS2B02G059100* and *TraesCS2D02G045600* were identified and designated *TaPT1-2B* and *TaPT2-2D*, respectively [19]. Notably, the coding sequences (CDS) of these three homologous genes (*TaPT1-2B*, *TaPT2-2D*, and *TaPT3-2D*) share 80.6% nucleotide sequence identity (Figure S1).

**Figure 1:**
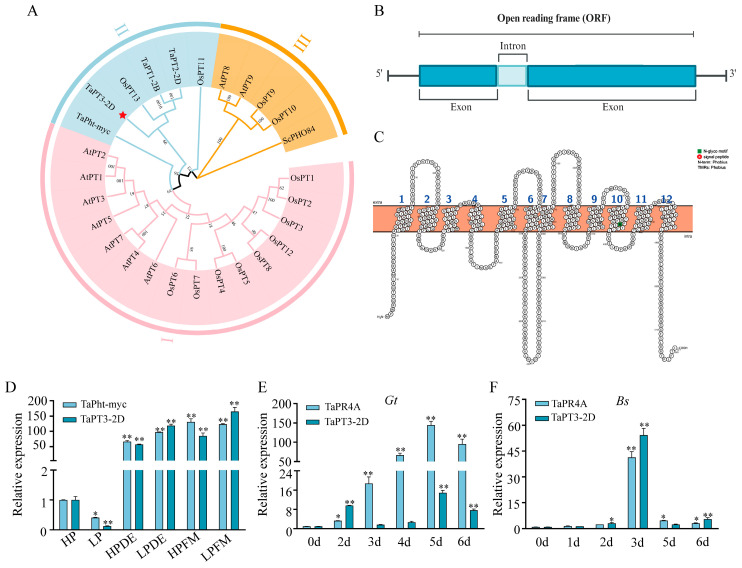
Characterization of TaPT3-2D. (**A**) A neighbor-joining (N-J) phylogenetic tree of phosphate transporters. Clade stability was evaluated by bootstrap resampling (n = 1000 replicates). (**B**) Gene structure of TaPT3-2D. (**C**) Transmembrane topology of TaPT31-7A predicted using Protter. (**D**) *TaPT3-2D* expression in arbuscular mycorrhiza-colonized wheat roots under different phosphate (Pi) regimes (42 days post inoculation). (**E**) *TaPT3-2D* expression in *Gaeumannomyces tritici*-infected roots. (**F**) *TaPT3-2D* expression in *Bipolaris sorokiniana*-infected roots. Asterisks indicate significant differences from the control according to one-way ANOVA followed by an independent-samples Dunnett’s post hoc test (* *p* ≤ 0.05; ** *p* ≤ 0.01).

Phylogenetic analysis incorporating previously characterized rice PHTs (Table S1) demonstrated that TaPT1-2B, TaPT2-2D, TaPT3-2D, TaPht-myc (a mycorrhizal-specific PHT1 protein), and two rice PHT1 members (OsPT11 and OsPT13) cluster within the same phylogenetic group (Figure 1A). The AM-specific or AM-inducible transporters OsPT11 and OsPT13 are known to mediate the development of AM symbioses and Pi acquisition [29]. Notably, TaPT3-2D and OsPT13 represent a distinct subcluster.

TaPT1-2B, TaPT2-2D, and TaPT3-2D share high protein sequence similarity (76.89%) (Figure S2) and exhibit significantly greater homology with OsPT13 (72.64–81.94%) than with OsPT11 (60.38–62.83%) (Figure S3, Table S2). The *TaPT3-2D* gene contains one intron, and its 1566 bp CDS encodes a 521 aa protein (Figure 1B). The intron-exon boundary conforms to the canonical GT-AG splicing rule, as confirmed by reference annotation in WheatOmics using JBrowse. In silico analysis predicted that TaPT3-2D contains 12 transmembrane (TM) domains (Figure 1C).

### 2.2. Expression Profiling of TaPT3-2D and Its Homologous Genes

Transcript abundance of *TaPT3-2D* was markedly upregulated upon colonization by two AM fungi, with the strongest induction observed under low-Pi conditions in plants colonized by *Diversispora epigaea* and *Funneliformis mosseae*. This expression pattern supports the classification of *TaPT3-2D* as an AM-inducible phosphate transporter. Under low-Pi conditions, *TaPT3-2D* expression in *D*. *epigaea*- and *F. mosseae*-colonized plants significantly exceeded that of *TaPht-myc*, the first identified mycorrhizal-specific *PHT1* gene in wheat (Figure 1D). In contrast, expression of *TaPT1-2B* and *TaPT2-2D* was significantly repressed by AM colonization, particularly in plants colonized by *D. epigaea* (Figure S4A).

To examine whether the expression of these three *TaPHT* genes is also affected by pathogen infection, wheat roots were inoculated with *Bipolaris sorokiniana* (*Bs*) and *Gaeumannomyces tritici* (*Gt*). As a well-established marker of pathogen challenge, *TaPR4A* exhibits diagnostic expression patterns during infection. Following *Gt* inoculation, *TaPR4A* transcription increased progressively and peaked at 5 days post inoculation (DPI) before declining modestly at 6 DPI. Expression of *TaPT2-2D* and *TaPT3-2D* was induced at 2 DPI, peaked at 5 DPI, and decreased by 6 DPI, whereas *TaPT1-2B* was significantly induced only at 2 DPI (Figure 1E and Figure S4B). Following *Bs* infection, *TaPR4A* and *TaPT3-2D* were significantly upregulated at 3 DPI and declined thereafter (Figure S4C). In contrast, *TaPT1-2B* and *TaPT2-2D* exhibited strong induction at 5 and 6 DPI. Given the observed involvement of *TaPT3-2D* in both AM symbiosis and pathogenic fungal infection, we next sought to elucidate its biological function.

### 2.3. Subcellular Localization of TaPT3-2D

**Figure 2:**
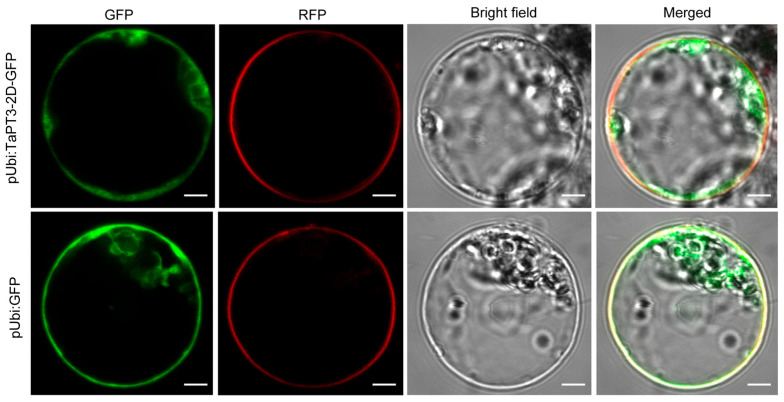
Subcellular localization of TaPT3-2D. Confocal microscopy images showing plasma membrane localization of TaPT3-2D-GFP compared with the diffuse cytosolic and nuclear distribution of GFP in rice protoplasts at 16 h post inoculation. Scale bars = 5 µm.

Given that phosphate transporters are typically localized to the plasma membrane, we assessed the subcellular localization of TaPT3-2D by transiently expressing a C-terminal GFP fusion under the control of the ubiquitin promoter in rice protoplasts. Consistent with the known properties of PHT1 proteins, TaPT3-2D-GFP fluorescence was observed exclusively at the plasma membrane, contrasting with the diffuse cytosolic and nuclear distribution of free GFP controls (Figure 2). These results therefore validate TaPT3-2D as a plasma membrane-localized phosphate transporter.

### 2.4. Complementation of TaPT3-2D in Yeast

**Figure 3:**
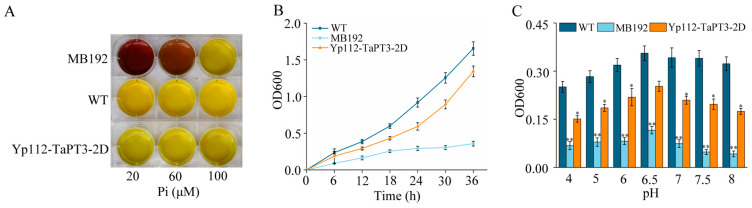
Functional validation of *TaPT3-2D* in phosphate uptake-deficient yeast. (**A**) Acid phosphatase activity assay of MB192, *TaPT3-2D*-complemented (Yp112-*TaPT3-2D*), and wild-type yeast grown under phosphate (Pi) limitation (20–100 μM). (**B**) Growth phenotypes in medium containing 60 μM Pi. (**C**) pH-dependent growth at 60 μM Pi (24 h). Asterisks indicate significant differences from the control according to one-way ANOVA followed by an independent-samples Dunnett’s post hoc test (* *p* ≤ 0.05; ** *p* ≤ 0.01).

The phosphate transporter activity of *TaPT3-2D* was evaluated using the yeast mutant MB192 transformed with p112A1NE-*TaPT3-2D*. Acid phosphatase activity staining revealed dose-dependent complementation at 20 and 60 μM Pi, suggesting that *TaPT3-2D* mediates Pi transport (Figure 3A). Growth kinetics in low-Pi YNB medium (60 µM) positioned *TaPT3-2D*-complemented MB192 cells between WT and mutant phenotypes, supporting partial functional recovery (Figure 3B). pH profiling identified pH 6.5 as optimal for *TaPT3-2D*-dependent Pi uptake, with complementation efficiency declining under both acidic and alkaline extremes (Figure 3C).

### 2.5. TaPT3-2D Is Required for AM Symbiosis and Pi Uptake

**Figure 4:**
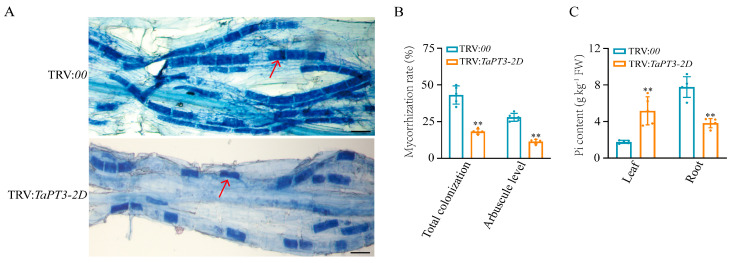
Silencing of *TaPT3-2D* impairs mycorrhizal colonization and alters phosphate distribution. (**A**) Representative images of trypan blue-stained wheat roots colonized by *Funneliformis mosseae* in control (TRV:00, **top**) and *TaPT3-2D*-silenced (TRV:*TaPT3-2D*, **bottom**) plants. Arrows indicate arbuscules. Scale bars = 20 µm. (**B**) Total mycorrhizal colonization rate and arbuscule abundance in control and *TaPT3-2D*-silenced plants at six weeks post-inoculation. Both parameters were significantly reduced in *TaPT3-2D*-silenced lines. (**C**) Pi concentrations in leaves and roots of control and *TaPT3-2D*-silenced plants. Silencing of *TaPT3-2D* decreased root Pi concentrations while increasing leaf Pi concentrations. Asterisks indicate significant differences from the control according to one-way ANOVA followed by an independent-samples Dunnett’s post hoc test (** *p* ≤ 0.01).

To characterize the role of *TaPT3-2D* in AM symbiosis, *TaPT3-2D* knockdown lines and control plants were inoculated with *F. mosseae*. Both the total colonization rate and arbuscule abundance were significantly lower in *TaPT3-2D* knockdown lines than in control plants. Mature arbuscules were observed in both control and virus-induced gene silencing (VIGS) plants, with no obvious differences in arbuscule morphology observed (Figure 4A,B). To further examine the role of *TaPT3-2D* in Pi transport, Pi concentrations were measured in roots and shoots. *TaPT3-2D*-silenced plants exhibited significantly lower Pi concentrations in roots, accompanied by increased Pi concentrations in shoots, suggesting that *TaPT3-2D* contributes to root-to-shoot Pi transport (Figure 4C).

### 2.6. Silencing of TaPT3-2D Increases Susceptibility to Fungal Pathogens

**Figure 5:**
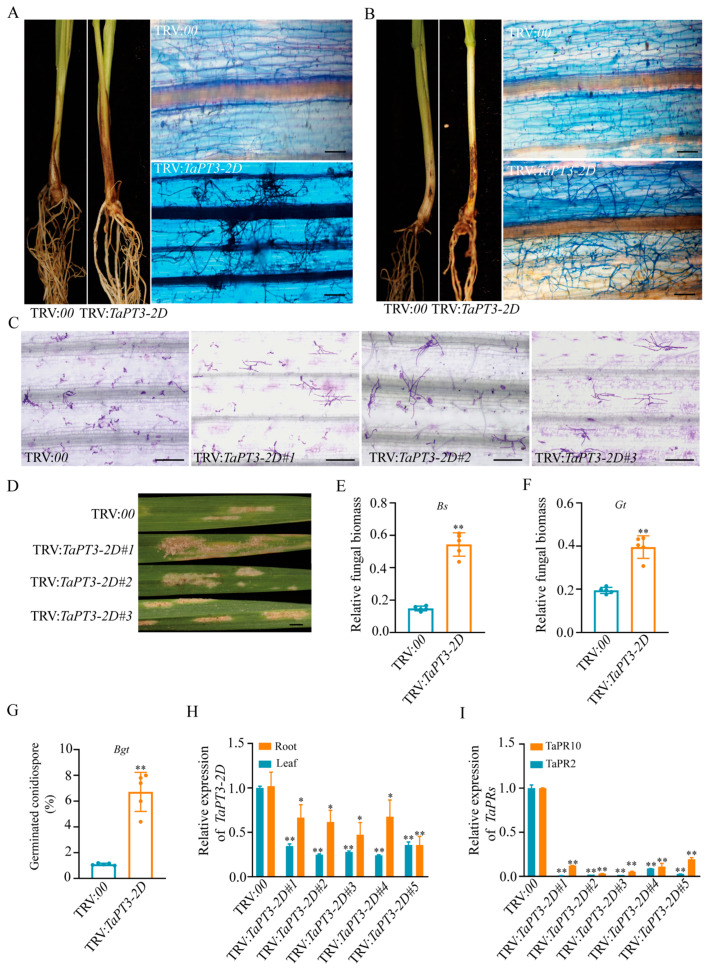
Silencing of *TaPT3-2D* enhances susceptibility to fungal pathogens and suppresses defense responses. (**A**) Disease symptoms and microscopic images of plants inoculated with *Bipolaris sorokiniana* (*Bs*) at 40 days post inoculation (DPI). (**B**) Disease symptoms and microscopic images of plants inoculated with *Gaeumannomyces tritici* (*Gt*) at 21 DPI. Microscopic (**C**) and macroscopic (**D**) images of leaves inoculated with *Blumeria graminis* f.sp. *tritici* (*Bgt*) at 60 h post inoculation (HPI). Relative fungal biomass of *Bs* (**E**) and *Gt* (**F**) in control and *TaPT3-2D*-silenced plants. (**G**) *Bgt* conidiospore germination rate on wheat leaves at 60 HPI. (**H**) Validation of *TaPT3-2D* silencing efficiency by qRT-PCR in roots and shoots. (**I**) Expression levels of defense-related genes *TaPR2* and *TaPR10* in control and silenced plants. Asterisks indicate significant differences from the control according to one-way ANOVA followed by an independent-samples Dunnett’s post hoc test (* *p* ≤ 0.05; ** *p* ≤ 0.01). Scale bars = 25 µm (**A**–**C**). Scale bars = 5 mm (**D**).

Given the strong induction of *TaPT3-2D* in response to pathogenic fungal infection, tobacco rattle virus (TRV)-based VIGS-mediated silencing of *TaPT3-2D* was performed to verify its role in wheat defense against pathogens. Following inoculation with the root pathogens *Gt* and *Bs* at 16 days after silencing, *TaPT3-2D*-VIGS lines exhibited accelerated disease progression. These plants showed significantly increased susceptibility to *Gt* at 21 DPI and to *Bs* at 40 DPI compared with control plants (Figure 5A,B). Consistently, both pathogens accumulated significantly more biomass in *TaPT3-2D*-silenced lines than in WT controls (Figure 5E,F).

Because the obligate biotroph *Blumeria graminis* f.sp. *tritici* (*Bgt*) employs a life strategy distinct from that of *Gt* and *Bs*, we further examined resistance to *Bgt* in *TaPT3-2D*-silenced wheat. The fourth leaves of *TaPT3-2D*-silenced and control (TRV:00) plants were harvested at 16 DPI and inoculated with *Bgt* conidiospores. Colonization efficiency at 60 h post-infection (HPI) was significantly higher in silenced lines compared to vector controls (Figure 5C–G). Macroscopic examination revealed extensive mycelial colonization of *TaPT3-2D*-silenced leaves (Figure 5D), consistent with microscopic analyses. Collectively, these results demonstrate that suppression of *TaPT3-2D* compromises wheat resistance to *Bgt* infection.

**Figure 6:**
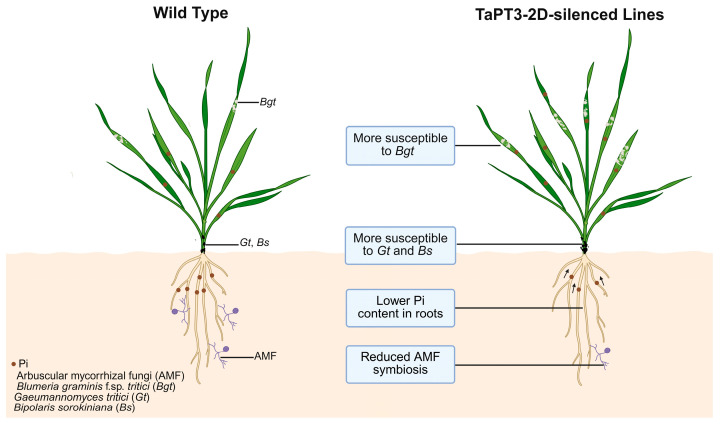
Proposed model of *TaPT3-2D* function in Pi uptake, arbuscular mycorrhizal (AM) symbiosis, and fungal pathogen resistance.

qRT-PCR validation confirmed efficient silencing of *TaPT3-2D*, with significantly reduced transcript abundance observed in both the roots and shoots of silenced plants compared to WT controls (Figure 5H). Concomitant with enhanced disease susceptibility, *TaPT3-2D*-silenced plants also exhibited marked downregulation of the defense-related genes *TaPR2* and *TaPR10* (Figure 5I). Together, these findings suggest that silencing of *TaPT3-2D* increases susceptibility to pathogens and impairs AM symbiosis and Pi uptake (Figure 6).

## 3. Discussion

### 3.1. TaPT3-2D Is a Novel Mycorrhizal-Induced PHT1 Similarly to OsPT13

Phylogenetic analysis revealed that TaPT3-2D and its two homologs form a monophyletic clade with three previously characterized AM-specific or AM-inducible phosphate transporters: OsPT13, OsPT11, and TaPht-myc (Figure 1A). Among these, TaPT3-2D exhibits the highest protein sequence similarity with OsPT13 (72.6%), followed by TaPht-myc (66.1%) and OsPT11 (62.3%) (Table S2). Notably, TaPT3-2D shares significantly higher sequence similarity with OsPT13 than with TaPht-myc, suggesting that TaPT3-2D is a functional ortholog of OsPT13 with a likely conserved role in mycorrhizal Pi transport.

Topological modeling further indicated that TaPT3-2D and OsPT13 both contain 12 TM domains, including a prominent hydrophilic loop linking TM6 and TM7, while both termini localize to the cytosol (Figure 1C) [29]. Previous studies have shown that OsPT13 is evolutionarily conserved exclusively in monocots, whereas OsPT11 exhibits cross-clade conservation across both monocots and dicots. Consistent with its mycorrhizal specificity, *OsPT13* is upregulated 14-fold in *Glomus intraradices*-inoculated roots [30]. In comparison, *TaPT3-2D* expression increased 57- to 165-fold in wheat roots colonized by *F. mosseae* or *D*. *epigaea* across different Pi regimes. The exceptionally strong induction of *TaPT3-2D* (>100-fold) by these AM fungi highlights its central role in AM-mediated Pi uptake in wheat. In contrast, neither *TaPT1-2B* nor *TaPT2-2D* was significantly induced by AM colonization, indicating functional divergence among these homologous genes (Figure S4A). Guided by these findings, we next sought to further characterize the role of *TaPT3-2D* in Pi acquisition and AM symbiosis.

### 3.2. TaPT3-2D Is a High-Affinity Phosphate Transporter Integral to Pi Assimilation and AM Symbiosis

To verify the role of *TaPT3-2D* in Pi transport, we employed the high-affinity phosphate transporter-deficient yeast strain MB192 (Δ*pho84*), a well-established system for functional validation of plant and fungal phosphate transporters [22,31,32,33]. Functional complementation with *TaPT3-2D* partially restored MB192 growth under low-Pi conditions (Figure 3), confirming its activity as a high-affinity phosphate transporter. The incomplete recovery of growth likely reflects limitations inherent to heterologous expression systems, including differences in transcriptional regulation, protein folding, and post-translational modification between plants and yeast [34].

VIGS provides a rapid and efficient approach for transient gene knockdown and functional analysis in plants, and TRV-based systems have been successfully applied in both monocots and dicots [35,36,37]. In *TaPT3-2D*-silenced wheat, Pi concentrations were significantly reduced in roots but increased in shoots, suggesting that *TaPT3-2D* contributes to root-to-shoot Pi transport. Several non-mutually exclusive mechanisms may explain this phenotype. First, silencing *TaPT3-2D* may induce a systemic Pi starvation response, leading to compensatory upregulation of other high-affinity Pi transporters (e.g., other *TaPHT1* members) or genes involved in phloem loading. Such responses could prioritize Pi allocation to shoots to sustain growth despite impaired root Pi acquisition or symplastic transport. Second, reduced AM colonization (Figure 4A,B) likely decreases Pi sink strength in roots, resulting in a greater proportion of acquired Pi being translocated to shoots. Finally, TaPT3-2D may play a direct role in Pi loading or unloading within roots, thereby modulating root-to-shoot translocation efficiency. Further studies examining the expression of other phosphate transporters in the *TaPT3-2D*-silenced background will be required to distinguish among these possibilities.

In addition to altered Pi distribution, *TaPT3-2D*-silenced plants exhibited significantly reduced total AM colonization rates and arbuscule abundance. Although these results demonstrate that *TaPT3-2D* is required for normal AM symbiosis, the functional consequences of this morphological impairment on nutrient exchange remain to be determined. Future studies using radioisotope tracers (e.g., ^33^P) will be essential to directly quantify mycorrhiza-mediated Pi uptake in *TaPT3-2D*-deficient plants. In parallel, analyses of carbon allocation, including the expression of symbiosis-associated sugar transporters and carbon flux to the fungal partner, will provide a more comprehensive assessment of symbiotic efficiency regulated by TaPT3-2D.

Although TRV-mediated VIGS enabled efficient initial functional characterization of TaPT3-2D in wheat, definitive validation of its biological role will require stable genetic lines. Future studies employing CRISPR/Cas9-mediated knockout and overexpression approaches will be essential to exclude potential confounding effects associated with transient silencing and to facilitate long-term investigations of Pi homeostasis, AM symbiosis, and immune responses.

### 3.3. TaPT3-2D Is Involved in the Regulation of Wheat Innate Immunity

TaPT3-2D is a member of the PHT1 family, one of the most extensively characterized phosphate transporter families in wheat. As expected, the majority of research on *PHT1* genes has focused on the roles of these genes in Pi uptake [20,21,22,38], whereas their functions in mycorrhizal symbiosis and plant immunity remain underexplored. In contrast, members of the PHT3 and PHT4 subfamilies have been more extensively examined for their roles in disease resistance [39,40,41]. For example, silencing of phosphate transporter *AtPHT4;1* enhances susceptibility to the virulent pathogen *Pseudomonas syringae* in *A. thaliana* [40]. Independent studies have established *AtPHT4;1* as a dual-function node coordinating pathogen defense and circadian regulation, and its expression is directly controlled by the core clock component CCA1 [42]. Similarly, silencing of the mitochondrial phosphate transporter *TaMPT* (a PHT3 family member) increases susceptibility to *Fusarium* head blight in wheat [39]. Consistent with these findings, we found that silencing *TaPT3-2D* increases wheat susceptibility to biotrophic, hemibiotrophic, and saprophytic fungi.

Notably, members of the same phosphate transporter family can exert contrasting effects on disease resistance. Although *AtPHT4;6* and *AtPHT4;1* both belong to the PHT4 family, silencing *AtPHT4;6* significantly enhances resistance to virulent *P. syringae* [41], underscoring functional divergence within this gene family. Similarly, *atpht1;4* mutants exhibit decreased susceptibility to the soil-borne bacterial pathogen *Ralstonia solanacearum* [43], while overexpression of the *PHT1* family gene *OsPT8* suppresses pattern-triggered immunity and attenuates resistance to rice blast (*M. oryzae*) and bacterial blight (*Xanthomonas oryzae* pv. *oryzae*) [44]. Collectively, although the effects of Pi transporters on disease susceptibility vary across pathosystems, these findings converge to support a functional link between PHT-mediated Pi uptake and plant immunity.

MicroRNAs (miRNAs) are also key regulators of Pi homeostasis, particularly miR399 and miR827. Overexpression of miR399 in *A. thaliana* and rice leads to Pi accumulation in leaves and increased susceptibility to *M. oryzae* [25,27]. Consistent with these reports, *TaPT3-2D*-silenced wheat exhibited elevated leaf Pi levels, enhanced pathogen susceptibility, and reduced AM symbiosis (Figure 4 and Figure 5). However, the mechanism by which plants integrate Pi status and microbial signals to coordinate defense and symbiosis remains elusive. Elucidating the molecular function of *TaPT3-2D* in Pi homeostasis and plant-microbe interactions will require protein–protein interaction analyses using yeast two-hybrid (Y2H), glutathione-S-transferase (GST) pull-down, bimolecular fluorescence complementation (BiFC), and firefly luciferase complementation imaging assays both in vivo and in vitro.

Although our data establish a link between *TaPT3-2D*, Pi homeostasis, AM symbiosis, and disease susceptibility, the underlying causal pathway remains to be resolved. The enhanced pathogen susceptibility observed in *TaPT3-2D*-silenced wheat may result from (i) direct disruption of Pi-dependent immune signaling, (ii) indirect effects caused by impaired AM symbiosis and the loss of symbiosis-associated defense benefits, or (iii) an independent, as-yet-unidentified role of *TaPT3-2D* in immune regulation. Future studies employing Pi supplementation assays in *TaPT3-2D* mutant backgrounds will be critical for distinguishing among these possibilities and determining whether altered immune responses primarily reflect changes in local Pi status or compromised symbiotic interactions.

Finally, although our results indicate that *TaPT3-2D* is required for full induction of defense-related genes such as *TaPR2* and *TaPR10*, the upstream signaling pathways affected by *TaPT3-2D* silencing remain unknown. Future work should systematically characterize the dynamics of key defense hormones, including SA, JA, and ethylene, and evaluate the expression of core pattern-triggered immunity (PTI) components in this genetic background. Such analyses will be essential to determine whether TaPT3-2D modulates immunity through specific hormonal pathways or by broadly influencing early immune perception and signaling, thereby defining a more precise molecular connection between phosphate transport and immune regulation.

## 4. Materials and Methods

### 4.1. Plant Materials and Fungal Inoculation

The Chinese winter wheat cultivar ‘Zhoumai 26’ was used for all experiments. Seedlings were transplanted into pots containing a sterilized mixture of soil and river sand at a 1:2 (*v*/*v*) ratio. To establish AM symbioses, autoclaved sand-based inocula containing 60 g of *F*. *mosseae* (Nicol. & Gerd, BGC XZ02A, isolated from the rhizosphere of Dangxiong, Tibet, China) or *D*. *epigaea* (BGC NM04B, formerly *Glomus versiforme*) were applied to 10 seedlings per treatment. Control plants received an equivalent amount of autoclaved inoculum. Both experimental and control plants were irrigated biweekly with half-strength Hoagland’s nutrient solution amended with either 5 μM (low-Pi) or 500 μM (high-Pi) KH_2_PO_4_. The experiment consisted of six treatment groups: high-Pi control (HP, 500 µM KH_2_PO_4_), low-Pi control (LP, 5 µM KH_2_PO_4_), high-Pi inoculated with *D*. *epigaea* (HPDE), low-Pi inoculated with *D*. *epigaea* (LPDE), high-Pi inoculated with *F. mosseae* (HPFM), and low-Pi inoculated with *F. mosseae* (LPFM). Tissue samples were collected at 42 DPI, immediately snap-frozen in liquid nitrogen, and stored at −80 °C for subsequent RNA extraction. The hemibiotrophic root rot pathogen *Bs*, provided by Henan Agricultural University, was cultured on potato dextrose agar (PDA) at 20 °C for 15 days. Ten-day-old seedlings were inoculated by applying *Bs* spore suspensions (4 × 10^4^ spores mL^−1^) to stem bases according to a previously described protocol [45]. Root samples were collected at 0, 1, 2, 3, 5, and 6 DPI for RNA extraction. A separate set of seedlings was inoculated with the necrotrophic pathogen *Gt* (formerly *Gaeumannomyces graminis* var. *tritici*), provided by Henan Academy of Agricultural Science, according to a previously described protocol [46]. Root tissues were collected at 0, 2, 3, 4, 5, and 6 DPI for RNA extraction. Pathogen-inoculated plants were maintained in a growth chamber at 70% relative humidity and 22 °C during the light period (16 h) and 15 °C during the dark period (8 h). The biotrophic fungal pathogen *Bgt* was maintained on ‘Aikang 58’ wheat at 22 °C during the light period (16 h) and 15 °C during the dark period (8 h). The pathogenesis-related 4 family gene *TaPR4A*, which is strongly induced by multiple fungal pathogens, was utilized as a molecular marker for pathogenicity [47].

Experiments involving AM colonization and pathogen challenge were conducted independently, and samples from each treatment were processed and analyzed separately to avoid confounding effects. Each treatment consisted of 10 individual plants, and all experiments were performed with three biological replicates.

### 4.2. Subcellular Localization of TaPT3-2D in Rice Protoplasts

The full-length *TaPT3-2D* CDS was cloned using KAPA HiFi HotStart ReadyMix (Roche, Basel, Switzerland) and inserted downstream of the *GFP* gene in the pUbi-GFP-GW vector via Gateway recombination, generating the *TaPT3-2D*-*GFP* fusion vector under the transcriptional control of the ubiquitin promoter. The recombinant construct was verified by Sanger sequencing and subsequently introduced into *Agrobacterium tumefaciens* strain GV3101. The empty GFP vector was transformed in parallel as a control. Rice protoplasts were isolated from 10-day-old rice seedlings, and protoplast transformation was performed using a previously published protocol [48]. The *TaPT3-2D*-*GFP* fusion construct or *GFP* control was introduced into protoplasts, which were then incubated in the dark at 28 °C for 16 h. Subcellular localization of the expressed proteins was examined using a confocal laser scanning microscope (Stellaris 8, Leica, Wetzlar, Germany), with FM4-64 staining employed as a plasma membrane marker. The green channel was excited at 488 nm with emission collected between 500–547 nm; the red channel was excited at 587 nm via a white-light laser line with emission collected between 600–632 nm. Images were assembled for presentation in Adobe Illustrator 2022.

### 4.3. RNA Isolation and Quantitative Real-Time PCR (qRT-PCR)

Total RNA was isolated using TRIzol reagent (Invitrogen, Carlsbad, CA, USA) following the manufacturer’s protocol. Genomic DNA contamination was removed by treatment with RNase-free DNase I (Takara Bio Inc., Kusatsu, Japan). Total RNA was reverse transcribed to synthesize cDNA using the PrimeScript RT Perfect Real Time Kit (Takara Bio Inc., Kusatsu, Japan). qRT-PCR was performed on a CFX^96^ Touch Real-Time PCR Detection System (Bio-Rad, Hercules, CA, USA) using SYBR^®^ Premix Ex Taq™ (Takara Bio Inc., Kusatsu, Japan). Relative gene expression levels were quantified using the 2^−ΔΔCt^ method [49]. Each sample was analyzed with three independent biological replicates and three technical repeats.

### 4.4. Functional Analysis of TaPT3-2D in Yeast

To ascertain the biological function of *TaPT3-2D*, the Pi uptake-deficient yeast mutant MB192 was transfected with the p112A1NE expression vector harboring *TaPT3-2D*, following a previously described method [50]. Briefly, the TaPT3-2D CDS was subcloned into the p112A1NE vector to generate the recombinant *TaPT3-2D*-p112A1NE construct, which was subsequently introduced into MB192 yeast. Next, *TaPT3-2D*-p112A1NE-harboring MB192 cells, WT cells, and untransformed MB192 cells were cultured in yeast nitrogen base (YNB) liquid medium. Upon reaching the logarithmic growth phase, cells were harvested by centrifugation and washed twice with Pi-free YNB. Next, the three yeast strains were separately inoculated into YNB liquid medium supplemented with 20, 60, or 100 µM KH_2_PO_4_ and incubated at 30 °C for 10 h. Bromcresol purple was employed as a pH indicator, with a medium color transition from brown-red to yellow signaling acidification as a correlate of yeast growth. For quantitative growth analysis, MB192, WT, and Yp112-TaPT3-2D yeast cells were cultured in YNB liquid medium supplemented with 60 μM KH_2_PO_4_. Cell cultures were harvested at six time points (6, 12, 18, 24, 30, and 36 h) to quantify the optical density at 600 nm (OD_600_). To examine the impact of pH on Pi uptake, each yeast strain was cultured in YNB liquid medium supplemented with 60 μM KH_2_PO_4_ and adjusted to different pH values (4, 5, 6, 6.5, 7, 7.5, and 8). Following a 24 h incubation period, OD_600_ was measured to assess cell growth. Each assay was performed with three independent biological replicates per strain.

### 4.5. VIGS Analysis of TaPT3-2D in Wheat

To validate the role of *TaPT3-2D* in AM symbiosis and Pi transport, gene silencing was performed using a TRV-based VIGS system. A highly conserved 300 bp segment of *TaPT3-2D* was chosen as the target region and amplified from ‘Zhoumai 26’ wheat. Subsequently, the In-Fusion HD Cloning Plus system was employed to perform recombination cloning, enabling the insertion of the *TaPT3-2D*-carrying pYL156 plasmid into the TRV vector to generate the TRV:*TaPT3-2D* construct. The empty pYL156 vector was similarly introduced into the TRV genome to generate the negative control TRV:00. To generate gene-silenced seedlings, ‘Zhoumai 26’ seeds were subjected to a modified whole-plant VIGS protocol [51], with unsilenced controls harboring the TRV:00 construct. At 16 DPI, total RNA was isolated from the roots and leaves of both VIGS-silenced and unsilenced plants to analyze *TaPT3-2D* expression.

### 4.6. Functional Verification of TaPT3-2D-Silenced Plants in Response to Fungal Infection

To verify the role of *TaPT3-2D* in the wheat response to fungal infection, 30 VIGS-silenced wheat seedlings were individually inoculated with either biotrophic symbiotic AM fungi, the hemi-biotrophic pathogen *Bs*, the necrotrophic pathogen *Gt*, or the biotrophic pathogen *Bgt*. VIGS-silenced and TRV:00 control plants were inoculated with *F. mosseae*. At 42 DPI, freshly harvested AM-colonized roots were collected and stained with trypan blue following a previously described protocol [52]. The frequencies of total and AM colonization were quantified for 30 root samples by magnifying intersections under a BX61 fluorescence microscope equipped with a 20× objective lens. Microscopic images of pathogens were similarly visualized using the same microscope configuration.

Pathogen inoculation procedures were performed as described in Section 4.1. For pathogen biomass quantification, qPCR was conducted using primers specific to the 18S rRNA genes of *Gt* (accession number: FJ771002) and *Bs* (accession number: KM066949). *TaActin* gene (accession number: KC775780) was utilized as the internal reference gene. Primers used for qRT-PCR are provided in Table S3. Finally, the total Pi concentration was quantified in fresh leaf and root samples from *TaPT3-2D*-silenced and control plants [53].

### 4.7. Statistical Analysis

Statistical analyses were performed using one-way analysis of variance (ANOVA) to evaluate differences in relative gene expression among treatments and time points, as well as phenotypic variation among genotypes. When ANOVA indicated significant effects (*p* < 0.05), Duncan’s multiple range test was used for post hoc comparisons. Least significant difference (LSD) values were calculated at the 95% and 99% confidence levels to support the analysis. Significant differences are indicated in the figures, and corresponding LSD values are provided in the figure legends. All statistical analyses were conducted using SPSS version 23.

## 5. Conclusions

In this study, we functionally characterized TaPT3-2D, an AM-inducible phosphate transporter in wheat. Heterologous expression in Δ*pho84* mutant MB192 yeast rescued Pi uptake deficiency, suggesting that *TaPT3-2D* functions as a phosphate transporter. VIGS-mediated suppression of *TaPT3-2D* attenuated pathogen resistance, reduced AM symbiosis, and decreased the Pi content in roots. Collectively, these findings demonstrate that TaPT3-2D serves as a key integrator of phosphate nutrition, symbiotic signaling, and disease resistance in wheat.

## Data Availability

The original contributions presented in this study are included in the article/Supplementary Material. Further inquiries can be directed to the corresponding author.

## Supplementary Materials

The following supporting information can be downloaded at https://www.mdpi.com/article/10.3390/plants15010118/s1. Figure S1. Nucleotide sequences of *TaPT1-2B*, *TaPT2-2D*, and *TaPT3-2D*. The red box shows the silenced region in *TaPT3-2D*. Figure S2. Amino acid sequences of TaPT1-2B, TaPT2-2D, and TaPT3-2D. Figure S3. PHT1 protein sequence similarity matrix. Figure S4. Expression patterns of *TaPT1-2B* and *TaPT2-2D*. (A) *TaPT1-2B* and *TaPT2-2D* expression levels in wheat roots under low/high Pi conditions at 42 days post inoculation with two arbuscular mycorrhizal species. (B) *TaPT1-2B* and *TaPT2-2D* expression levels in *Gaeumannomyces tritici* (Gt)-infected roots. (C) *TaPT1-2B* and *TaPT2-2D* expression levels in *Bipolaris sorokiniana* (*Bs*)-infected roots. Table S1. Accession numbers of PHT proteins for constructing phylogenetic tree of TaPT3-2D. Table S2 Identities of PHT proteins used for constructing the phylogenetic tree of TaPT3-2D. Table S3 Primers used in this study.
