# Supplementary figures and images for: Silencing of the Mycorrhiza-Inducible Phosphate Transporter TaPT3-2D in Wheat Enhances Pathogen Susceptibility and Impairs Arbuscular Mycorrhizal Symbiosis
Source: Plants (Basel). 2026 Jan 1;15(1):118. doi: 10.3390/plants15010118 (PMC12787379; doi:10.3390/plants15010118)

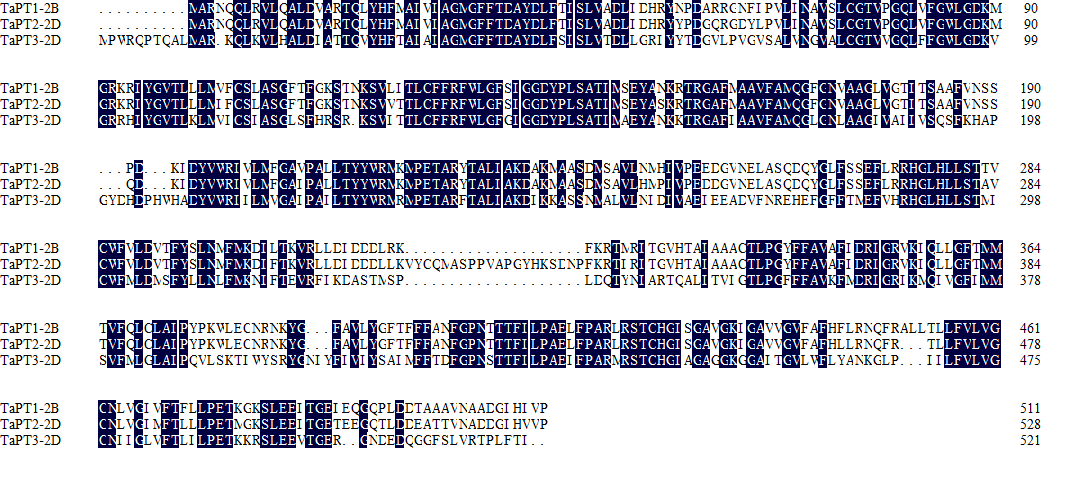

Supplement: Supplementary file 1 [file plants-15-00118-s001.zip › Figure S2╡░░╫▒╚╢╘.tif]

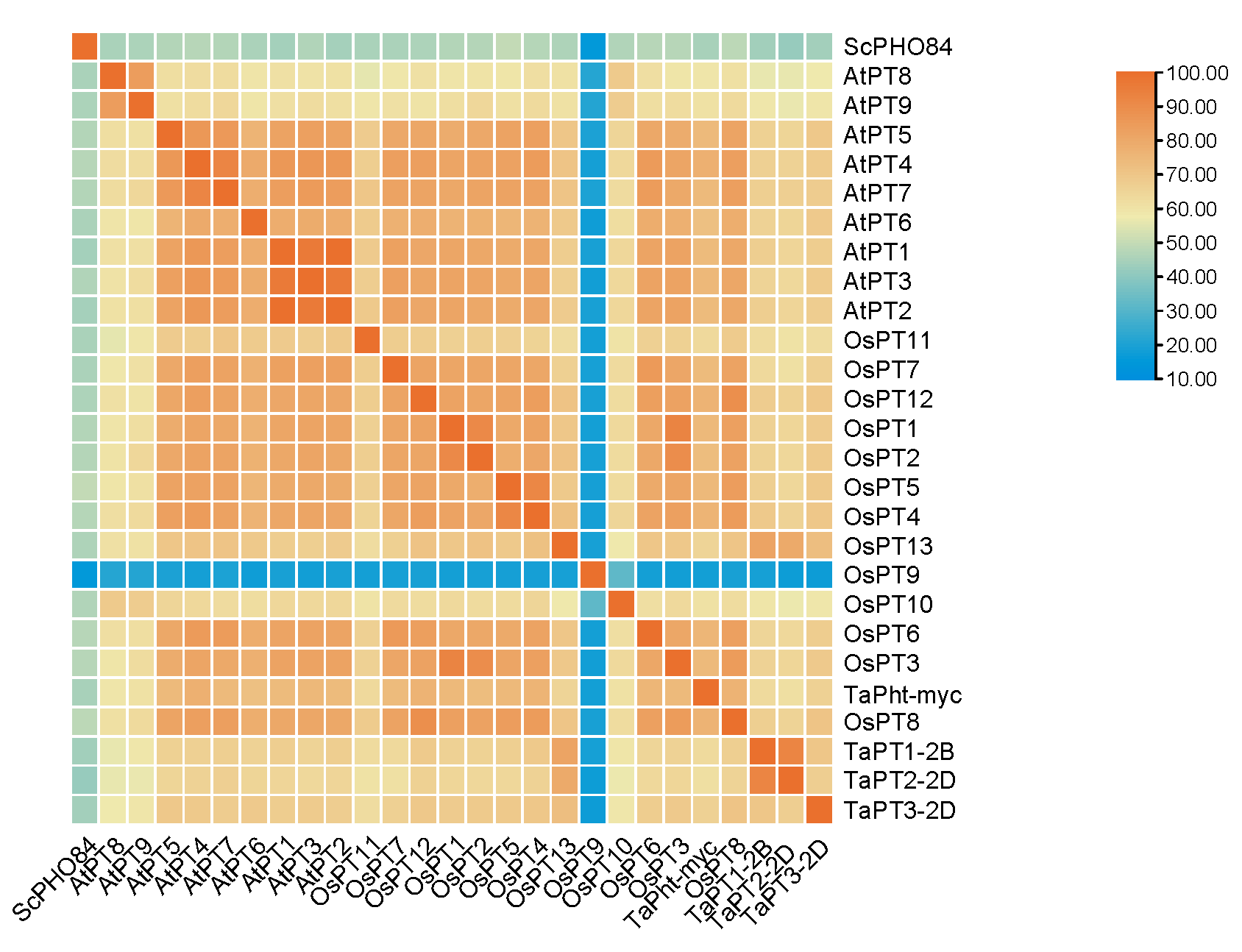

Supplement: Supplementary file 1 [file plants-15-00118-s001.zip › Figure S3.tiff]

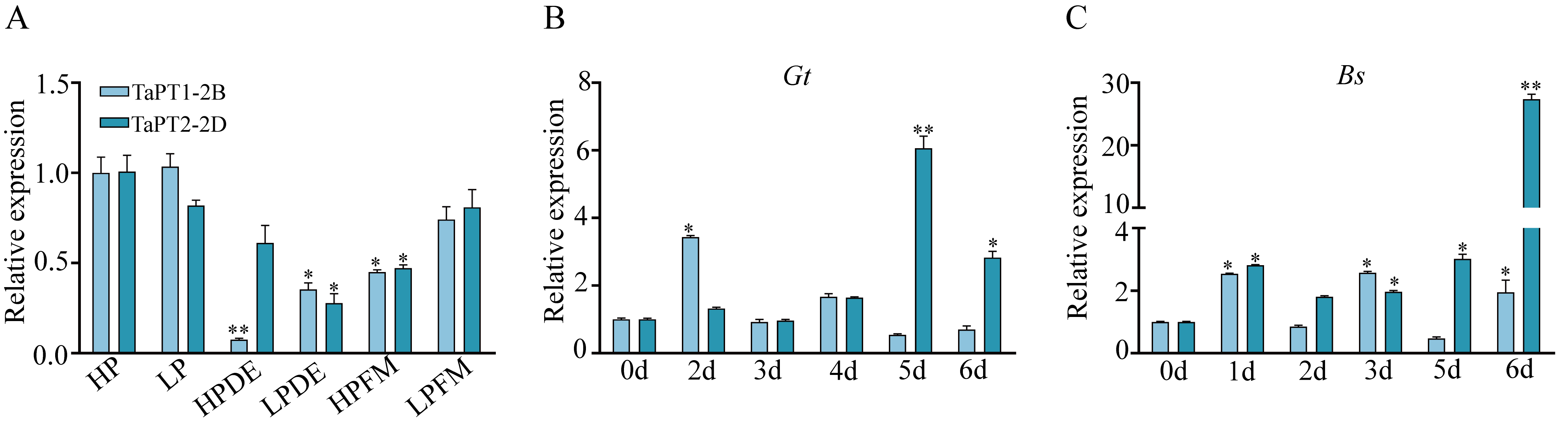

Supplement: Supplementary file 1 [file plants-15-00118-s001.zip › Figure S4 .tif]

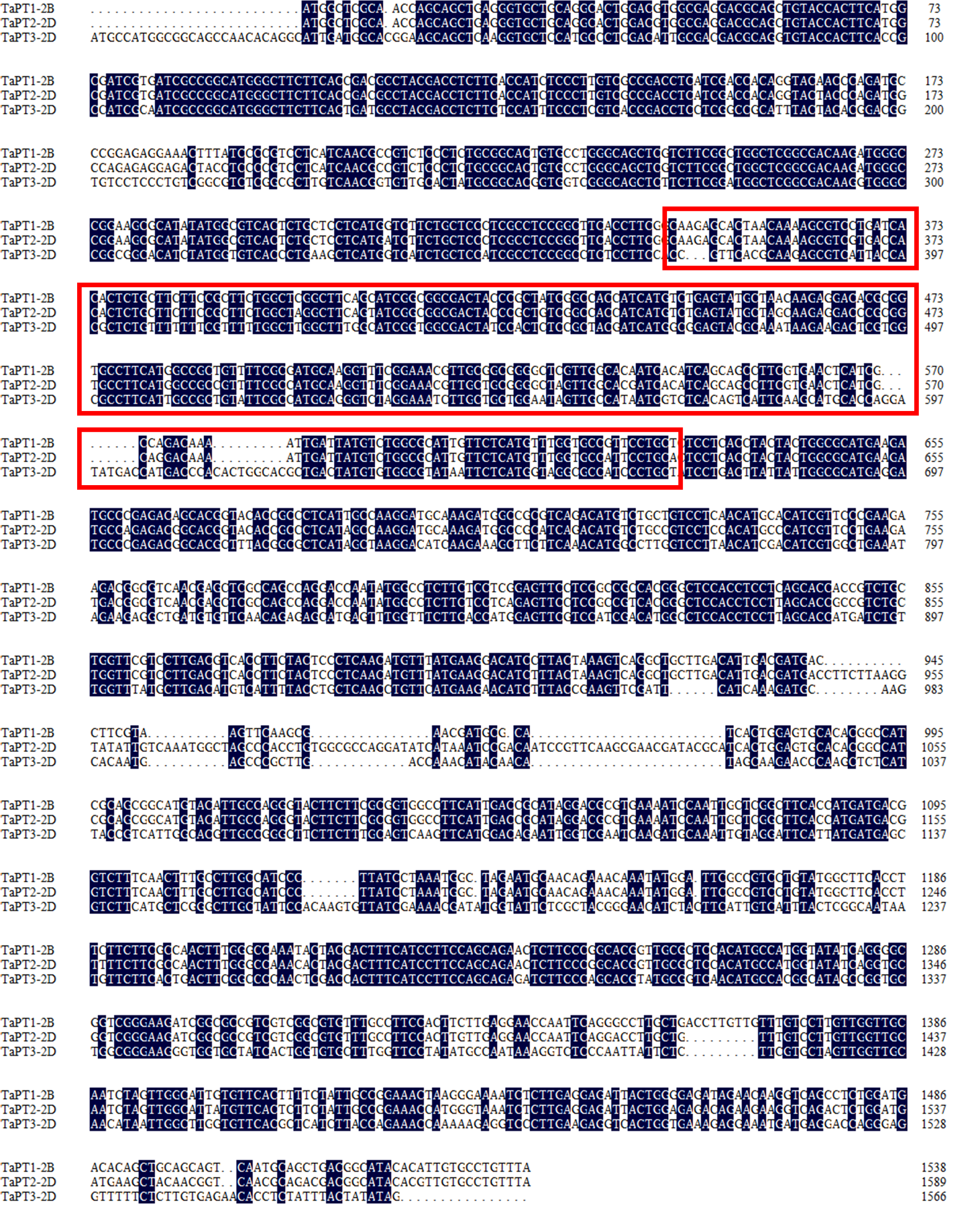

Supplement: Supplementary file 1 [file plants-15-00118-s001.zip › Figure S1.tif]
